# Survival after hypofractionation in glioblastoma: a systematic review and meta-analysis

**DOI:** 10.1186/s13014-020-01584-6

**Published:** 2020-06-08

**Authors:** Jane-Chloe Trone, Alexis Vallard, Sandrine Sotton, Majed Ben Mrad, Omar Jmour, Nicolas Magné, Benjamin Pommier, Silvy Laporte, Edouard Ollier

**Affiliations:** 1 Department of Radiation Oncology, Lucien Neuwirth Cancer Institute, 108 Bis, Avenue Albert Raimond, 42270 Saint-Priest-en-Jarez, France; 2 University Departement of Research and Teaching, Lucien Neuwirth Cancer Institute, Saint-Priest-en-Jarez, France; 3 Department of Neurosurgery, University Hospital, Saint-Etienne, France; 4 SAINBIOSE U1059, Jean Monnet University, Saint-Etienne, France

**Keywords:** glioblastoma, radiotherapy, survival outcome, hypofractionation, methodology, meta-analysis, review

## Abstract

**Background:**

Glioblastoma multiforme (GBM) has a poor prognosis despite a multi modal treatment that includes normofractionated radiotherapy. So, various hypofractionated alternatives to normofractionated RT have been tested to improve such prognosis. There is need of systematic review and meta-analysis to analyse the literature properly and maybe generalised the use of hypofractionation. The aim of this study was first, to perform a meta-analysis of all controlled trials testing the impact of hypofractionation on survival without age restriction and secondly, to analyse data from all non-comparative trials testing the impact of hypofractionation, radiosurgery and hypofractionated stereotactic RT in first line.

**Materials/Methods:**

We searched Medline, Embase and Cochrane databases to identify all publications testing the impact of hypofractionation in glioblastoma between 1985 and March 2020. Combined hazard ratio from comparative studies was calculated for overall survival. The impact of study design, age and use of adjuvant temozolomide was explored by stratification. Meta-regressions were performed to determine the impact of prognostic factors.

**Results:**

2283 publications were identified. Eleven comparative trials were included. No impact on overall survival was evidenced (HR: 1.07, 95%CI: 0.89-1.28) without age restriction. The analysis of non-comparative literature revealed heterogeneous outcomes with limited quality of reporting. Concurrent chemotherapy, completion of surgery, immobilization device, isodose of prescription, and prescribed dose (depending on tumour volume) were poorly described. However, results on survival are encouraging and were correlated with the percentage of resected patients and with patients age but not with median dose.

**Conclusions:**

Because few trials were randomized and because the limited quality of reporting, it is difficult to define the place of hypofactionation in glioblastoma. In first line, hypofractionation resulted in comparable survival outcome with the benefit of a shortened duration. The method used to assess hypofractionation needs to be improved.

## Introduction

Glioblastoma multiforme (GBM) is the most aggressive malignant primary brain tumour with a median overall survival of 12-15 months [1]. The prognosis is poor despite a multi modal treatment that includes normofractionated radiotherapy. The Stupp protocol, is composed of complete surgical resection followed by concurrent chemoradiation (6 weeks) plus adjuvant chemotherapy [2]. Failure to complete standard radiation therapy is associated with decreased survival [3]. Moreover, 80% of relapses happen in an already irradiated zone [4]. As a result, alternatives to the Stupp protocol have been tested to decrease relapse rate. Moderate hypofractionation (dose >2.2 Gy/fraction) aimed at reducing the duration of treatment in elderly patients. However, it seemed that it might produce both an increase in cancer cells death and a decrease in the tumour repopulation [5]. Clinical trials using extreme hypofractionation (>6 Gy/fraction) for first line treatment were conducted [6]. The total dose could be delivered either in a few fractions (hypofractionated stereotactic radiotherapy (hSRT)) or in just one (stereotactic radiosurgery (SRS)), as boost after normofractionated radiotherapy, in order to maximize the biological effects of hypofractionation [5, 7, 8].

Multiple trials based on variations of fractionation (moderate or extreme hypofractionation) and/or of radiotherapy technique (SRS, hSRT) have been carried out [9]. Yet, most studies were retrospective or single-arm phase I/II trials with few patients included. In addition to important heterogeneities in radiation characteristics, patients were treated for various disease status (first line treatment or relapse). The results of such studies were contradictory which made the impact of fractionation on glioblastoma prognosis hard to figure out [8, 10–12]. A comprehensive analysis of all data about the impact of radiation characteristics on GBM prognosis has never been carried out.

The aim of this study was first to perform a meta-analysis of all comparative trials testing the impact of hypofractionation on survival. Secondly, we analysed data about all non-comparative trials testing the impact of hypofractionation (non-stereotactic hypofractionated radiotherapy, hSRT and radiosurgery) in first line.

## Materials and Methods

Requests were performed in the Medline, Embase and Cochrane databases to identify all publications testing the impact of hypofractionation in glioblastoma between 1985 (first trial) and 2020. In case of several publications for the same trial, only the most recent data was taken into account. The latest update was performed in March 2020. All reviews on the topic were also studied to ensure that major studies had not been omitted.

### Study selection

Two of the authors (JCT and EO) independently evaluated studies for possible inclusion. Studies were eligible for inclusion if patients had high grade glioma treated with hypofractionation in first line, regardless of the radiotherapy technique: non-stereotactic hypofractionated (dose>2.2Gy/fraction), hSRT (1-5 fractions, dose per fraction > 6Gy with increased accuracy of patient’s positioning and radiation ballistics) or radiosurgery (mono fractionated hSRT >10 Gy).

Studies were excluded in the following cases: if primary was not a brain tumor, if treatment did not include radiotherapy, if fractionation was not tested, in case concurrent treatment was changed between 2 treatment arms, in case of ongoing study or non-human study, in case of comments/letters/guideline publications.

#### Meta-analysis

The following MeSH terms were used: ‘high grade glioma’, ‘glioblastoma’, ‘hypo fractionation’, ‘hypo fractionated’, ‘stereotactic’, ‘radiosurgery’, ‘clinical trials’. A first selection was carried out and based on title and abstract. Then, eligible articles were selected on full text and then reviewed. Only phase II and III trials testing two different fractionations and featuring overall survival data were analysed.

#### Analysis of non-comparative trials

The following MeSH terms were used: ‘high grade glioma’, ‘glioblastoma’, ‘hypofractionated’, ‘stereotactic’, ‘radiosurgery’, ‘radiation therapy’, ‘radiotherapy’. A first selection was conducted based on title and abstract. Then, eligible articles were selected on full text and reviewed. Only trials featuring overall survival data were analysed.

### Data collection

Data were independently extracted by two of the authors (JCT and EO). In the event of discrepancies between the reviewers, a consensus was reached by discussion.

For each selected trial, the following data was collected: study characteristics (author’s name, year of publication, number of included patients, number of patient in each arm), design of the study, patient characteristics (age, extent of surgical resection (subtotal/gross total *vs* biopsy)), tumour characteristics (grade, volume, O^6^-methylguanine-DNA methyltransferase (MGMT) promoter methylation status), radiation characteristics (volume, dose, technique and type of machine, fractionation, duration of whole treatment, dose prescription to isodose line), additional or concurrent treatments (surgery, chemotherapy, targeted therapy, immune therapy), survival outcome.

### Statistical analysis

For the analysis of comparative clinical trial, a fixed-effects model based on the logarithm of the hazard ratio (HR) weighted by the inverse of the variance was used for combining results from the individual data. Statistical heterogeneity among studies was explored using Cochrane’s Q statistic, study consistency being quantified by means of the I^2^ statistic [13]. In case of significant heterogeneity (*P*-value less than 0.10) with no clear explanation for this, a random-effect model was used for data analysis [14]. For the association, a *P*-value less than 0.05 was considered statistically significant. The results of the meta-analysis are presented graphically. The effect size expressed as HR with the corresponding 95% confidence interval (CI) was included. HR=1 indicates the treatments made no difference. HR<1 indicates that hypofractionated radiotherapy was better and HR>1 indicates that control (normo-fractionation) was better. The results were considered statistically significant when the 95% confidence interval did not contain 1. Effect size was estimated globally, according to trial design (randomized *vs* non-randomized studies), median age (<65 years *vs* ≥65 years) and concomitant treatment (temozolomide *vs* no temozolomide).

Regarding non-comparative clinical trials, a classical analysis of median survivals was impossible due to the quality of statistical reporting. Indeed, confidence intervals were missing in most articles. By weighting studies by their respective sample size, we provided the value of pooled median survivals for descriptive purpose only. A 95% confidence interval was calculated using nonparametric bootstrap. To explore the impact of study level value of prognostic factors (age, proportion of patient with surgical resection, radiation therapy dose) on survival, we performed fixed-effect meta-regression on logarithm of median survivals. In these analyses, studies were also weighted using their respective sample size.

All statistical analyses were performed using R statistical software, version 3.3.1 with the meta packages (version 4.7).

## Results

### Meta-analysis of controlled trials testing the impact of hypofractionation on survival (first line treatment).

**Fig. 1:**
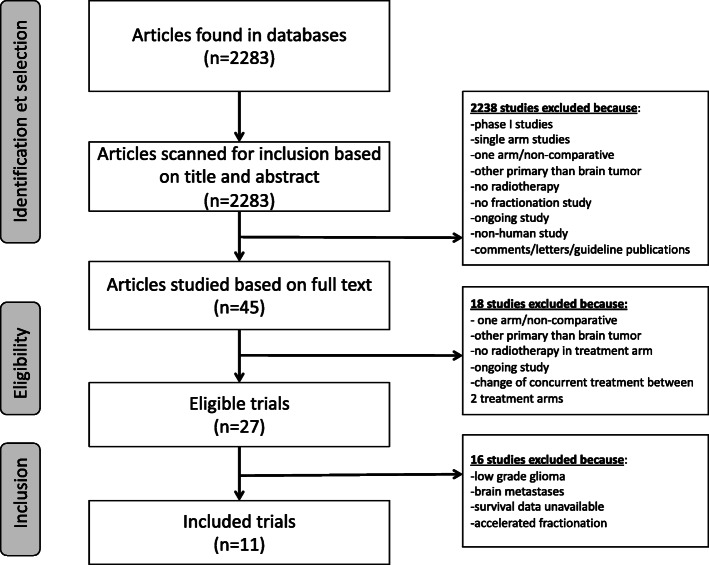
Flow chart about selection of controlled trials for meta-analysis

**Fig. 2:**
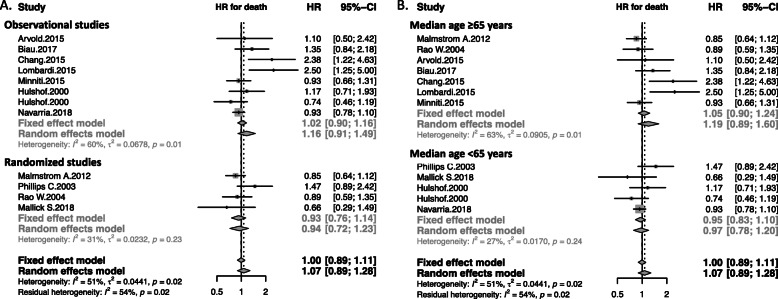
Meta-analysis of controlled trials analysing by design (**a**) (observational *vs*. randomized studies) and by median age (**b**) (<65 years *vs* ≥65 years) testing hypofractionation on newly diagnosed high-grade glioma or glioblastoma. The size of the symbols is proportional to the number of included patients

Four randomized controlled trials (two phase III, two phase II) [15–18] and 7 observational studies (8 arms) [19–25] were identified (flow-chart: see Fig. 1). Studies compared normofractionation with hypofractionation. They were all open-label trials published between 2000 and 2018. A total of 1738 patients were included with a median age of 70 (range: 45-75). Seven studies included only patients over 65 years. The primary tumor was a newly diagnosed GBM in 10 studies and high grade glioma in one [17]. MGMT promoter methylation status was analysed in 7 studies. Patients with previous radiation therapy treatment (*i.e.* second line patients) were excluded. Data about extent of surgical resection was available in all studies with a median of 69% patients with subtotal/gross total resection. The primary endpoint was overall survival in all studies. The objective of randomized controlled trials was to prove either superiority (2 study) or non-inferiority (2 studies [15, 17]) of experimental arm. Radiotherapy was 3D conformal normo-fractionated radiotherapy in all “standard treatment” arms (1.8-2 Gy per fraction, 1 fraction a day, 5 fractions a week). In experimental arms, hypofractionated radiotherapy was based on fractions of 2.667-5 Gy, 3-5 fractions per week. Concurrent chemotherapy was associated to radiation in seven studies. Characteristics of studies are listed in Additional file 1. The meta-analysis showed no significant difference in overall survival (HR: 1.07, 95%CI: 0.89-1.28) (Fig. 2). Analysis by design (randomized *vs* observational studies) revealed a non-significant trend toward overestimation of hypofractionation effect in observational studies (ratio of HR= 1.22 95%CI 0.81-1.82, p for interaction = 0.34) (Fig. 2a). Stratification of the meta-analysis on the median age (<65 years *vs* ≥65 years) revealed no significant interaction between hypofractionation effect and median age (p for interaction = 0.37) (Fig. 2b).

Stratification of the meta-analysis on the use of concomitant temozolomide chemotherapy revealed no significant interaction between hypofractionation effect and the use of concomitant temozolomide chemotherapy (p for interaction = 0.32) (Additional file 2).

### Analysis of non-comparative trials testing the impact of hypofractionation (>3Gy/fraction) on survival (first line treatment).

Twenty one non-comparative studies assessed the impact of hypofractionation in newly diagnosed glioblastoma patients in 22 treatment arms [4, 6, 8, 11, 12, 20, 26–40] (Additional file 3). Outcomes were compared with the ones of the Stupp trial, which is currently considered as the reference in the management of first-line glioblastoma [2]. Most studies were single arm Phase I or II trials. The mean number of included patient per arm was 33. The radiotherapy technique was heterogeneous: 14 trials were based on non-stereotactic hypofractionated radiotherapy (intensity modulated radiotherapy=6, three-dimensional conformal radiotherapy=8) and 7 trials were based on hSRT. Out of the hSRT studies, 3 combined hSRT with a normofractionated radiotherapy (delivering 44-60 Gy). The prescription isodose was defined in 4 out of the 7 hSRT studies and ranged from 80% isodose to 100% isodose. The dose per fraction delivered in hSRT trials ranged from 4 to 20 Gy, with a mean total dose of 36 Gy. In the trials based on “non-stereotactic” hypofractionation (*i.e.* non-hSRT), the dose per fraction ranged from 2.4 to 8.5 Gy, with a mean total dose of 40.1 Gy. MGMT promoter methylation status was analysed in 8 studies.

#### Chemoradiation trials

**Fig. 3:**
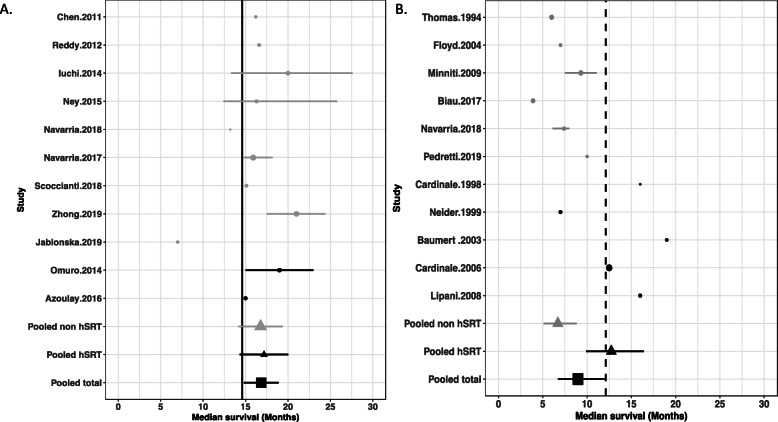
Median overall survival in chemoradiation trials based on hypofractionated radiotherapy (**a**) and in trials based on exclusive hypofractionated radiotherapy (**b**) (grey dots: non-stereotactic techniques (IMRT, 3D-CRT): *vs.* black dots: stereotactic radiotherapy). Basis (vertical line): chemoradiation arm of the Stupp trial (4A) and exclusive normofractionated radiotherapy arm of the Stupp trial (4B). The size of the symbols is proportional to the number of included patients. **hSRT** : hypofractionated stereotactic radiotherapy; **non hSRT**: non-stereotactic techniques (IMRT, 3D-CRT)

**Fig. 4:**
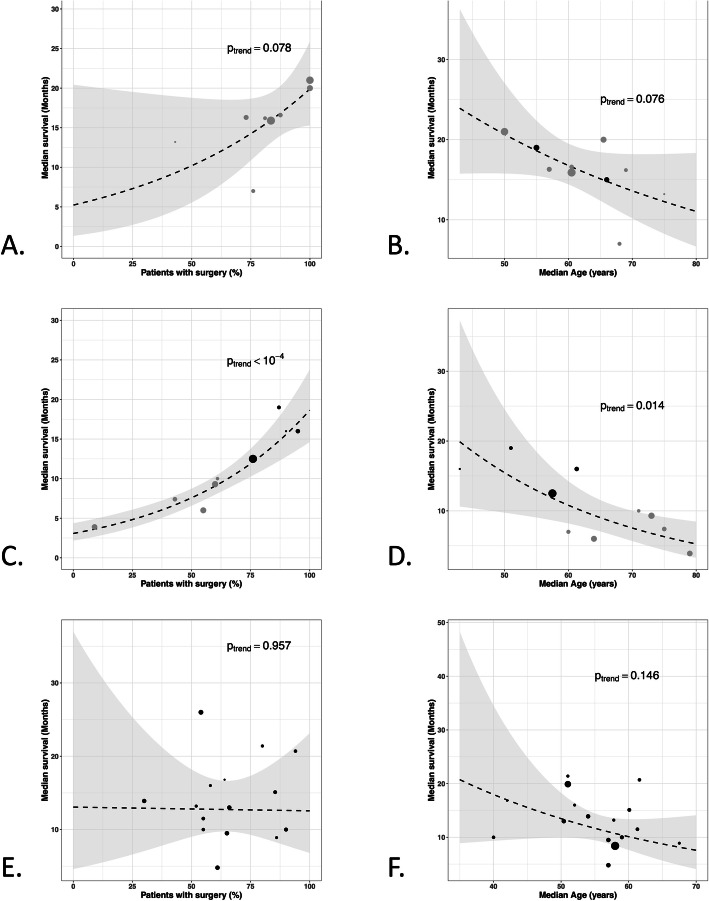
Relationship between median survival within each study and percentage of patients with subtotal/gross total resection (**a**) and median age (**b**) in chemoradiation trials; percentage of patients with subtotal/gross total resection (**c**) and median age (**d**) in exclusive hypofractionation trials; percentage of patients with subtotal/gross total resection (**e**) and median age (**f**) in radiosurgery trials. (in **a**, **b**, **c**, and **d**: grey dots: non-stereotactic techniques (IMRT, 3D-CRT): *vs.* black dots: stereotactic radiotherapy)

Among the 22 treatment arms, 11 tested a concurrent chemoradiation (temozolomide or temozolomide-bevacizumab) [11, 26–34, 40]. Radiotherapy was hSRT in 2 arms whereas 9 arms used conventional techniques. Five studies included patients with age ≥ 65 years and median age was 65.5 years (range 50-75 years). Data about extent of surgical resection (subtotal/gross total *vs* biopsy) was available in 8 studies. The mean radiation dose was 60 Gy, in fractions of 2.4-8.5 Gy. Normofractionated radiotherapy was never added. The median overall survival of the experimental arms (hypofractionated radiotherapy plus chemotherapy) was of 16.8 months (95%CI 14.6-19.1). The chemoradiation Stupp arm achieved a median overall survival of 14.6 months (95%CI 13.2-16,8). Median overall survival of 9 of the 11 experimental arms was superior to the one obtained in the chemoradiation arm of the Stupp trial (range: 7-21 months). Outcomes did not differ between hSRT and non-stereotactic hypofractionated radiotherapy (17.2 months (95%CI 14.4-20.0) *vs* 16.8 months (95%CI 14.2-19.3)) respectively. Results of the statistical analyses are given in Fig. 3a. Median survival seems to be correlated with the percentage of surgical resection (*p* = 0.08) and with patients median age (*p* = 0.08) (Fig. 4a and b) and there is no correlation with median dose (*p* = 0.56) (Additional file 4A).

#### Exclusive radiotherapy trials

Hypofractionated radiotherapy was exclusively performed in eleven arms [4, 6, 8, 12, 20, 35–39]. The mean total dose was 35.9 Gy (range: 20-52.5) in fractions of 2.7-20 Gy. Five arms were based on hSRT, delivering a mean dose of 28-50 Gy. In three arms, a normofractionated radiotherapy was added (44-60 Gy) [6, 38, 39]. Four studies included patients with age ≥ 65 and median age was 64 (range 43-79 years). Data about the extent of surgical resection (subtotal/gross total *vs* biopsy) was available in 9 studies. The median overall survival of the hypofractionated arms was 8.9 months (95%CI 6.7-11.9). The median overall survival in trials based on non-stereotactic hypofractionation was of 6.7 months (95%CI 5.1-8.8). The median overall survival of the hSRT arms was 12.7 months (95%CI 9.9-16.4). In hSRT trials when normofractionnated radiotherapy was associated to stereotactic radiation median overall survival was ≥ 16 months [6, 12, 39]. The “exclusive radiation” Stupp arm achieved a median overall survival of 12.1 months (95%CI 11.2-13). Results are plotted in Fig. 3b. Median survival seems to be significantly correlated with the percentage of surgical resection (*p* < 0.001) and with patients median age (*p* = 0.014) (Fig. 4c and d). There is no correlation with median dose (*p* = 0.278) (Additional file 4B). The observed difference in survival between of non-stereotactic hypofractionation and hSRT trials is certainly driven by confounding factors as patients included in hSRT trials are older and have less surgery than patients from non-stereotactic hypofractionation trials (Fig. 4c and d).

### Analysis of radiosurgery in first line.

**Fig. 5:**
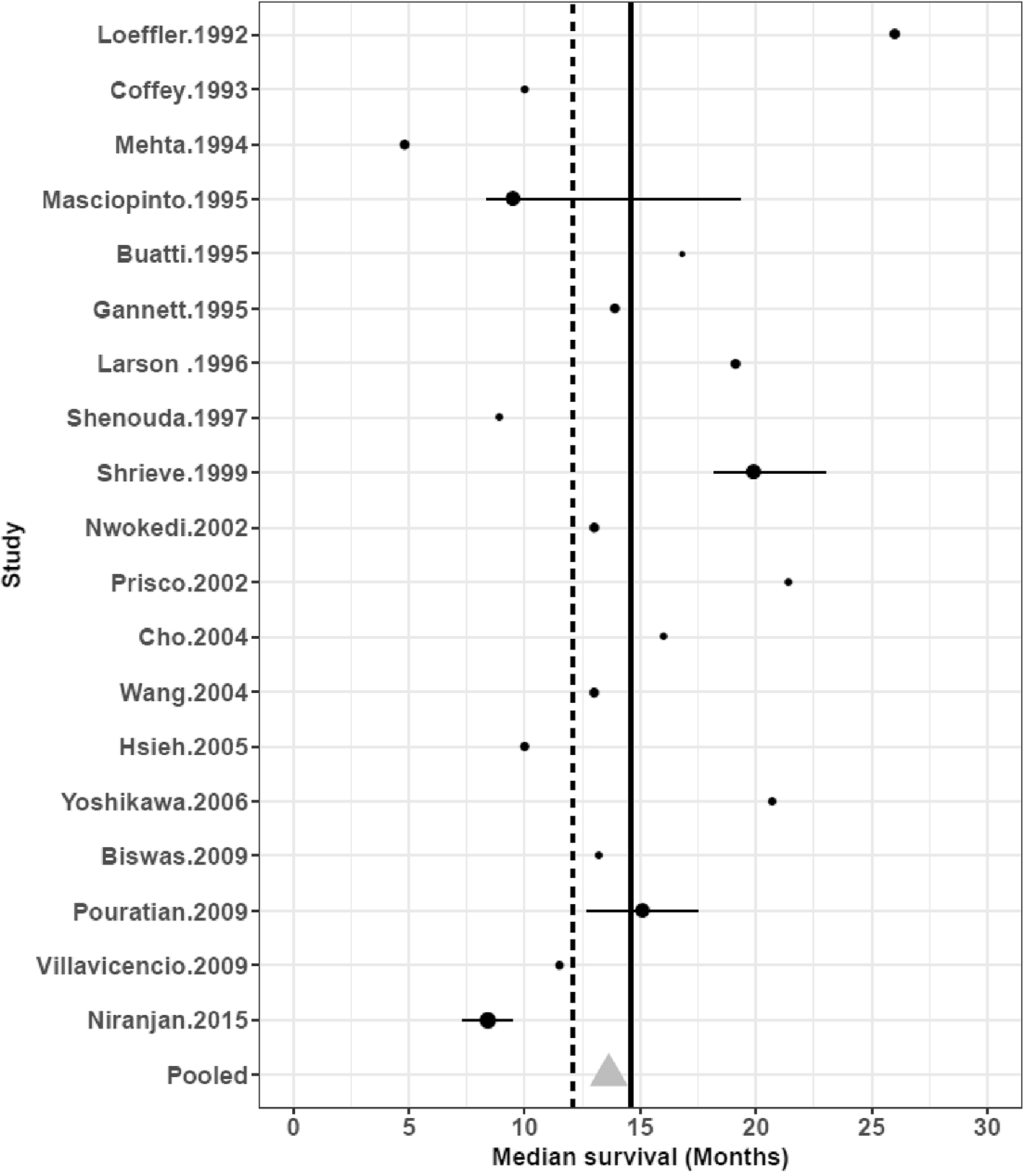
Median overall survival in non-randomized trials based on SRS as first line treatment of newly diagnosed glioblastoma. Vertical line (dotted): exclusive normofractionated radiotherapy arm of the Stupp trial, Vertical line (full line): concurrent chemoradiation arm of the Stupp trial. The size of the symbols is proportional to the number of included patients

Twenty SRS studies were identified [7, 41–59]. They were mainly retrospective and included an average of 32 patients (Additional file 5). Only one study was a prospective randomized controlled phase III trial [59]. Normofractionated radiotherapy was associated to SRS in 19 studies and delivered a mean additional dose of 60 Gy. SRS was employed as boost associated to normofractionated radiotherapy and not as exclusive treatment in the vast majority of studies. Only one study included patients with age ≥ 65 [46] and median age was 58 (range 40-67.5). Data about extent of surgical resection (subtotal/gross total *vs* biopsy) was available in 15 studies. The mean tumor volume was 15.4 cc. In any study MGMT promoter methylation status was analysed. The mean dose of SRS was 14.5 Gy (range : 10-20.3 Gy). The prescription isodose was described in 14 studies and ranged from the 50% to the 100%. The prescription of chemotherapy before SRS was heterogeneous. Data about chemotherapy were poorly reported and could therefore not be taken into account in the present analysis. The median OS with SRS was 12.5 months (95%CI 9.3-15.7). Results of non-randomized trials are plotted in Fig. 5. Overall survival was superior to the chemoradiation arm in the Stupp protocol in 8 SRS arms (15.1-26 months). Yet, this difference has to be interpreted cautiously as the analysis does not take into account the confounding effect of prognosis factor. In these studies, the range of the mean SRS dose was similar to the other trials: 13.8 Gy (range: 10-20.3 Gy). No dose-effect relationship was evidenced (*p* = 0.622) (Additional file 4C) and median survival does not seem to be not correlated with the percentage of surgical resection (*p* = 0.957) or patients median age (*p* = 0.146) (Fig. 4e and f). Conversely, the date when the study was published seemed to influence the treatment efficacy. Indeed, the lowest median overall survival was found in trials published before 1996. This is probably due to SRS technical evolutions as well as the increasing use of chemotherapy, surgery and supportive care treatments. Finally, the trial with the longest survival (26 months) included 14 anaplastic astrocytomas out of the 37 high grade gliomas.

## Discussion

The meta-analysis of the eleven comparative trials about hypofractionated radiotherapy as first line treatment in GBM patients shows no significative difference compared to standard radiotherapy both in all patients including the elderly. Therefore, hypofractionation radiotherapy may appear as an acceptable alternative for patients whose poor condition prevented them from having normofractionated radiotherapy.

However, some studies show that a significant proportion of elderly GBM patients still received standard chemo-radiotherapy [60]. Hypofractionated RT may be used more widely given the results of this meta-analysis but a high powered non inferiority randomized trial would be necessary to definitively validate this strategy. Although non-significant, our analysis shows a potential diverging estimation of hypofractionation effect between randomized and observational studies. In meta-analyses only based on observational studies, we should be careful in the interpretation of results that can mistakenly conclude that hypofractionated RT could be not a safety option for all patients [9] and may risk to skew therapeutic decision-making.

With concomitant temozolomide, hypofractionated RT seems to be comparable to normofractionated RT. These results are consistent with non-comparative trials studying non-stereotactic hypofractionated RT. In non-comparative trials, overall survival seems to be correlated with median age and the number of patients with surgical treatment with or without concomitant temozolomide. Prospective randomised studies assessing the role of hSRT as first line treatment are missing. RTOG 9305 is the only phase III study that assessed the role of radiosurgery. The use of an additional boost in radiosurgery showed that overall survival was not improved [59]. Although the results of retrospective trials remained encouraging, its place is still to be defined. Similarly, it is difficult to draw a conclusion about the role of radiosurgery as first line treatment as almost only retrospective phase I/II trials with contradictory results have been carried out so far and SRS was employed as boost associated to normofractionated radiotherapy and not as an exclusive treatment.

Besides, the present study concludes that the quality of reporting in published trials needs to be improved. Although they are major survival predictors, concurrent anticancer treatments were little or not mentioned in non-comparative trials. Moreover, the completion of surgery was rarely detailed as for MGMT promoter methylation status. Radiotherapy technique was also poorly described since isodose of prescription was rarely reported (Additional files 2 and 3). Finally, the 95% confidence intervals of overall survival were rarely available, which makes pooled statistical analysis impossible.

This study has some limitations. First, due to poor reporting of MGMT promoter methylation status, its impact on overall survival has not been investigated. Secondly, it would be interesting to consider the impact of hypofractionated RT or re-irradiation on quality of life [61]. Finally, it would be useful to compare the different short-course radiation therapy regimens.

Thus, such heterogeneity in treatments limits the authors’ conclusions. It appears necessary first, to define clear, precise and standardised procedures and secondly to come to an agreement about dose prescription. Finally, the quality of reporting of information from randomised and non-randomised trials must also be improved and and it is time for current guidelines to be followed [62, 63].

## Conclusion

Because very few trials were randomised and because the quality of reporting in non-comparative trials was limited, it is difficult to clearly define the place of hypofactionation in glioblastoma. In first line, non-stereotactic hypofractionation, especially with concomitant temozolomide, seems to be comparable to normofractionated RT with short-time benefits. Survivals after hSRT and SRS in first line were heterogeneous so a reliable conclusion cannot be drawn. Finally, the method used to assess innovating techniques such as hSRT and SRS definitely needs improving. Besides, the fact that they were never compared to the current gold standard treatment limits the level of evidence of such trials. Yet, conducting prospective randomised trials is not easy. Indeed, the number of eligible patients is high and indications of hSRT and radiosurgery are rare. Thus, prospective phase II trials may be considered but the same quality of methodology as in phase III randomised trials should be used so as to ensure the results can be validated.

## Supplementary information

**Additional file 1: Table 1.** Characteristics of randomized controlled studies testing hypofractionation, included in the meta-analysis.

**Additional file 2: Figure 1.** Meta-analysis of controlled trials analysing by using concomitant temozolomide (no temozolomide *vs* temozolomide) testing hypofractionation on newly diagnosed high-grade glioma or glioblastoma. The size of the symbols is proportional to the number of included patients.

**Additional file 3: Table 2.** Characteristics of non-randomized trials assessing the outcome of hypofractionation in newly diagnosed glioblastoma or high grade glioma.

**Additional file 4: Figure 2.** Relationship between median survival within each study and median dose in gray in chemoradiation trials (A); in exclusive hypofractionation trials (B); in radiosurgery trials (C).

**Additional file 5: Table 3*.*** Trials studying the efficacy of radiosurgery in GBM.

## Data Availability

Not applicable

## Abbreviations

GBM: glioblastoma multiforme
RT: radiation therapy
hSRT: hypofractionated stereotactic radiotherapy
SRS: stereotactic radiosurgery
RC3D: Three-dimensional conformal radiotherapy
IMRT: intensity modulated radiotherapy
MGMT: O^6^-methylguanine-DNA methyltransferase
HR: Hazard Ratio
