# Supplementary material for: Survival after hypofractionation in glioblastoma: a systematic review and meta-analysis
Source: Radiat Oncol. 2020 Jun 8;15:145. doi: 10.1186/s13014-020-01584-6 (PMC7278121; doi:10.1186/s13014-020-01584-6)
Supplement: Supplementary file 1 — Additional file 1: Table 1. Characteristics of randomized controlled studies testing hypofractionation, included in the meta-analysis. [file 13014_2020_1584_MOESM1_ESM.docx]

**Table S1**: Characteristics of randomized controlled studies testing hypofractionation, included in the meta-analysis

| **Author,**  **year** | **Primary**  **tumor** | **Trial design** | **Nb of included patients** | **Median age (years)** | **Experimental scheme**  -Technique  -Total dose  -Fractionation | **Standard scheme**  -Technique  -Total dose  -Fractionation |
| --- | --- | --- | --- | --- | --- | --- |
| Phillips,  2003 [17] | High grade glioma | Randomized  Phase 2 | 68 | 58.5 | -3D-CRT  -35Gy  -3.5Gy/fr., 1fr. a day, 5fr. a week | -3D-CRT  -60Gy  -2Gy/fr., 5 fr. a week |
| Roa,  2004 [15] | GBM | Randomized  Phase 3 | 100 | 71 | -3D-CRT  -40Gy  -2.67Gy/fr., 1fr. a day, 5fr. a week | -3D-CRT  -60Gy  -2Gy/fr., 5 fr. a week |
| Malmstrom  2012 [16] | GBM | Randomized  Phase 3 | 223 | 70 | -3D-CRT  -30Gy  -5Gy/fr., 1fr. a day, 3fr. a week | -3D-CRT  -60Gy  -2Gy/fr., 5 fr. a week |
| Mallick  2018 [18] | GBM | Randomized  Phase 2 | 89 | 45 | -3D-CRT  -60Gy  -3Gy/fr., 1fr. a day, 5fr. a week | -3D-CRT  -60Gy  -2Gy/fr., 5 fr. a week |
| Arvold  2015 [19] | GBM | Observational study | 91 | 73 | -3D-CRT  -35Gy  -3.5Gy/fr., 1fr. a day, 5fr. a week | -3D-CRT  -60Gy  -2Gy/fr., 5 fr. a week |
| Biau  2017 [20] | GBM | Observational study | 70 | 75 | -3D-CRT  -40Gy  -2.67Gy/fr., 1fr. a day, 5fr. a week | -3D-CRT  -60Gy  -2Gy/fr., 5 fr. a week |
| Chang  2015 [21] | GBM | Observational study | 129 | 70 | -3D-CRT  -35Gy  -3.5Gy/fr., 1fr. a day, 5fr. a week | -3D-CRT  -60Gy  -2Gy/fr., 5 fr. a week |
| Lombardi  2015 [22] | GBM | Observational study | 237 | 71 | -3D-CRT  -40Gy  -2.67Gy/fr., 1fr. a day, 5fr. a week | -3D-CRT  -60Gy  -2Gy/fr., 5 fr. a week |
| Minniti  2015 [14] | GBM | Observational study | 243 | 70 | -3D-CRT  -40Gy  -2.67Gy/fr., 1fr. a day, 5fr. a week | -3D-CRT  -60Gy  -2Gy/fr., 5 fr. a week |
| Hulshof  2000 [23] | GBM | Observational study | 107 | 60 | -3D-CRT  -40Gy  -5Gy/fr., 1fr. a day, 5fr. a week | -3D-CRT  -66Gy  -2Gy/fr., 5 fr. a week |
| Hulshof  2000 [24] | GBM | Observational study | 114 | 60 | -3D-CRT  -28Gy  -7Gy/fr., 1fr. a day, 4fr. a week | -3D-CRT  -66Gy  -2Gy/fr., 5 fr. a week |
| Navarria  2018 [25] | GBM | Observational study | 267 | 61 | -3D-CRT  -60Gy  -4Gy/fr., 1fr. a day, 5fr. a week | -3D-CRT  -60Gy  -2Gy/fr., 5 fr. a week |

Abbreviations : fr. : fraction; 3D-CRT : Three dimensional conformal radiotherapy ; GBM : glioblastoma multiforme
