# Supplementary material for: Survival after hypofractionation in glioblastoma: a systematic review and meta-analysis
Source: Radiat Oncol. 2020 Jun 8;15:145. doi: 10.1186/s13014-020-01584-6 (PMC7278121; doi:10.1186/s13014-020-01584-6)
Supplement: Supplementary file 2 — Additional file 2: Figure 1. Meta-analysis of controlled trials analysing by using concomitant temozolomide (no temozolomide vs temozolomide) testing hypofractionation on newly diagnosed high-grade glioma or glioblastoma. The size of the symbols is proportional to the number of included patients. [file 13014_2020_1584_MOESM2_ESM.docx]

**Figure 1.** Meta-analysis of controlled trials analysing by using concomitant temozolomide (no temozolomide *vs* temozolomide) testing hypofractionation on newly diagnosed high-grade glioma or glioblastoma. The size of the symbols is proportional to the number of included patients.
