# Supplementary material for: Survival after hypofractionation in glioblastoma: a systematic review and meta-analysis
Source: Radiat Oncol. 2020 Jun 8;15:145. doi: 10.1186/s13014-020-01584-6 (PMC7278121; doi:10.1186/s13014-020-01584-6)
Supplement: Supplementary file 3 — Additional file 3: Table 2. Characteristics of non-randomized trials assessing the outcome of hypofractionation in newly diagnosed glioblastoma or high grade glioma. [file 13014_2020_1584_MOESM3_ESM.docx]

**Table 2**: Characteristics of non-randomized trials assessing the outcome of hypofractionation in newly diagnosed glioblastoma or high grade glioma

**Abbreviations** : Nb=number ; GBM : glioblastoma multiforme ; G III : grade III glioma ; hSRT : hypofractionated stereotactic radiotherapy ; RC3D : Three-dimensional conformal radiotherapy ; IMRT : intensity modulated radiotherapy ; NR : Not reported ; Bvcz : bevacizumab ; TMZ : temozolomide, cc : cubic centimeter)

**Data about high grade gliomas were available

| **Author** | **Year** | **Phase** | **Design** | **Nb of patients** | **Histology** | **Median age (years)** | **Median tumor volume (cc)** | **Radiation technique** | **Associated normo-fractionated radiotherapy** | **Dose (Number of fraction *dose per fraction)** | **Mean Dose** | **Prescription isodose** | **Concurrent chemotherapy** | **Median overall survival (months)** |
| --- | --- | --- | --- | --- | --- | --- | --- | --- | --- | --- | --- | --- | --- | --- |
| Cardinale [6] | 1998 | Phase I | Single arm | 10 | GBM* | 43 | 15.9 | hSRT | Yes (44 Gy) | 3 * 12 Gy | 36 Gy | 100% | No | 16 |
| Cardinale [41] | 2006 | Phase II | Single arm | 76 | GBM | 57.5 | NR | hSRT | Yes (50 Gy) | 4*5-7 Gy | NR | 100% | No | 12.5 |
| Nieder [39] | 1999 | Phase I | Single arm | 19 | GBM | NR | NR | hSRT | No | 5*5-7 Gy | NR | NR | No | 7 |
| Lipani [12] | 2008 | Retrospective | Single arm | 20 | GBM | 61.3 | 86 | hSRT | No | 20 to 41Gy in 1 to 8 fractions | 35 Gy | 80% | No | 16 |
| Baumert [40] | 2003 | Phase I/II | Single arm | 15 | GBM* | 51 | NR | hSRT | Yes (60 Gy) | 2*10 Gy or 5*4 Gy | 20 Gy | 90% | No | 19 |
| Floyd [36] | 2004 | Phase I | Single arm | 18 | GBM | 60 | NR | IMRT | No | 10*5 Gy | 50 Gy | NR | No | 7 |
| Thomas [8] | 1994 | Phase II | Single arm | 38 | 28 GBM, 4 G III** | 64 | NR | RC3D | No | 6*5 Gy | 30 Gy | NR | No | 6 |
| Minniti [37] | 2009 | Phase II | Single arm | 43 | GBM | 73 | NR | RC3D | No | 6*5 Gy | 30 Gy | NR | No | 9.3 |
| Pedretti [38] | 2019 | Phase II | 2 randomized treatment arms | 14 | GBM | 71 | NR | RC3D | No | 6*5 Gy | 30 | NR | No | 10 |
| Biau [20] | 2017 | Phase II | 3 non- randomized treatment arms | 34 | GBM | 79 | NR | RC3D | No | 15*2.67 Gy | 40 | NR | No | 3.9 |
| Navarria [31] | 2018 | Phase II | Single arm | 22 | GBM | 75 | NR | RC3D | No | 15*3.5 Gy | 52.5 | NR | No | 7.4 |
| Omuro [34] | 2014 | Phase II | Single arm | 40 | GBM | 55 | NR | hSRT | No | 6*6 Gy | 36 Gy | NR | Bvcz + TMZ | 19 |
| Azoulay [35] | 2016 | Phase I/II | Single arm | 30 | GBM | 66 | 26.8 | hSRT | No | 5*5-8 Gy | NR | NR | TMZ | 15 |
| Chen [27] | 2011 | Phase I | Single arm | 16 | GBM | 69 | NR | IMRT | No | 20*3 Gy, 15*4 Gy, 12*5 Gy, 10*6 Gy | 60 Gy | 90% | TMZ | 16.2 |
| Iuchi [29] | 2014 | Phase II | Single arm | 46 | GBM | 65.5 | NR | IMRT | No | 8*8.5 Gy | 68 Gy | NR | TMZ | 20 |
| Ney [30] | 2015 | Phase II | Single arm | 30 | GBM | 57 | NR | IMRT | No | 10*6 Gy | 60 Gy | 90% | Bvcz + TMZ | 16.3 |
| Reddy [28] | 2012 | Phase I | Single arm | 24 | GBM | 60.5 | NR | IMRT | No | 10*6 Gy | 60 Gy | 90% | TMZ | 16.6 |
| Scoccianti [32] | 2018 | Phase II | Single arm | 24 | GBM | NR | NR | RC3D | No | 15*4.5 Gy | 67.5 | NR | TMZ | 15.1 |
| Jablonska [11] | 2019 | Phase II | Single arm | 17 | GBM | 68 | NR | RC3D | No | 15*3.3 Gy | 50 | NR | TMZ | 7 |
| Zhong [33] | 2019 | Phase II | Single arm | 80 | GBM | 50 | NR | RC3D | No | 27*2.37 Gy | 64 | NR | TMZ | 21 |
| Navarria [31] | 2018 | Phase II | Single arm | 8 | GBM | 75 | NR | RC3D | No | 15*3.5 Gy | 52.5 | NR | TMZ | 13.2 |
| Navarria [42] | 2017 | Phase II | Single arm | 97 | GBM | 60.5 | 79 | IMRT | No | 15*4 Gy | 60 | 95 | TMZ | 15.9 |
