# Supplementary material for: Survival after hypofractionation in glioblastoma: a systematic review and meta-analysis
Source: Radiat Oncol. 2020 Jun 8;15:145. doi: 10.1186/s13014-020-01584-6 (PMC7278121; doi:10.1186/s13014-020-01584-6)
Supplement: Supplementary file 4 — Additional file 4: Figure 2. Relationship between median survival within each study and median dose in gray in chemoradiation trials (A); in exclusive hypofractionation trials (B); in radiosurgery trials (C). [file 13014_2020_1584_MOESM4_ESM.docx]

**Figure 2**: Relationship between median survival within each study and median dose in gray in chemoradiation trials (A); in exclusive hypofractionation trials (B); in radiosurgery trials (C).

(in A. and B.: grey dots: non-stereotactic techniques (IMRT, 3D-CRT): *vs.* black dots: stereotactic radiotherapy)
