# Supplementary material for: Survival after hypofractionation in glioblastoma: a systematic review and meta-analysis
Source: Radiat Oncol. 2020 Jun 8;15:145. doi: 10.1186/s13014-020-01584-6 (PMC7278121; doi:10.1186/s13014-020-01584-6)
Supplement: Supplementary file 5 — Additional file 5: Table 3. Trials studying the efficacy of radiosurgery in GBM. [file 13014_2020_1584_MOESM5_ESM.docx]

**Table 3***.* Trials studying the efficacy of radiosurgery in GBM.

Abbreviations : Nb=number ; GBM : Glioblastoma multiforme ; AA : anaplastic astrocytoma ; NR : Not reported ; GK : GammaKnife

*Data about high grade gliomas were available

| **Author** | **Year** | **Phase** | **Design** | **Nb of**  **patients** | **Histology** | **Median age**  **(years)** | **Median Tumor volume (cc)** | **Associated normo-fractionated radiotherapy (dose)** | **Machine** | **Dose (Gy)** | **Mean dose (Gy)** | **Isodose of prescription (%)** | **Concurrent treatment** | **Median overall survivall (months)** |
| --- | --- | --- | --- | --- | --- | --- | --- | --- | --- | --- | --- | --- | --- | --- |
| Souhami [61] | 2004 | Phase III | Randomized, 2 arms | 203 | GBM | 56 | NR | Yes (60Gy) | GK or Linac | 15-24 | NR | 50 to 90 | No | 13.5 |
| Coffey [7] | 1993 | Phase I | Single arm | 18 | AA GBM | 40 | NR | Yes (NR) | NR | 12-18 | 15 | 50 | No | 10 |
| Loeffler [43] | 1992 | Phase I/II | Single arm | 37 | 14 AA, 23 GBM | NR | 4,8 | Yes (59,4Gy) | GK | 10-20 | 12 | NR | No | 26 |
| Masciopinto [44] | 1995 | Phase I/II | Single arm | 31 | GBM | 57 | 16,4 | Yes (50-66Gy) | Linac | 10-20 | 11,7 | isocenter | No | 9,5 |
| Mehta [45] | 1994 | Phase I/II | Single arm | 31 | GBM | 57 | 17,4 | Yes (54-60Gy) | Linac | 10-20 | 12 | 72,5 | No | 4,8 |
| Buatti [46] | 1995 | Phase I | Single arm | 11 | 5AA, 6 GBM | 42 | 14 | Yes (60Gy) | NR | 10-15 | 12,5 | 80 | No | 16,8 |
| Gannett [47] | 1995 | Phase I | Single arm | 30 | 11 AA, 19 GBM | 54 | 24 | Yes (59,4Gy) | Linac | 0,5-18 | 10 | 100 | No | 13,9 |
| Shenouda [48] | 1997 | Phase I | Single arm | 14 | GBM | 67.5 | NR | Yes (60Gy) | NR | 20 | 20 | 90 | No | NR |
| Nwokedi [49] | 2002 | retrospective | Single arm | 31 | GBM | 50.4 | 25 | Yes (59,7Gy) | GK | 10-28 | 17,1 | 50 | No | 13 |
| Cho [50] | 2004 | retrospective | 2 arms | 14 | GBM | 52 | 27 | Yes (60Gy) | X-Knife | 10-18 | 10,5 | 86 | No | 16 |
| Hsieh [51] | 2005 | retrospective | Single arm | 25 | GBM | 59 | 23,6 | Yes (60Gy) | GK | 15-32 | 12 | 50 | No | 10 |
| Yoshikawa [52] | 2006 | retrospective | Single arm | 18 | GBM* | 61.6 | 19,1 | Yes, in 50% (44,5 Gy) | CyberKnife | 13,9-26,4 | 20,3 | 90 | possible | 20,7 |
| Biswas [53] | 2009 | retrospective | Single arm | 15 | GBM | 57.8 | 13,2 | Yes (60Gy) | Novalis | 6-20 | 13 | 80 | No | 13,2 |
| Pouratian [54] | 2009 | retrospective | Single arm | 22 | GBM | 60.1 | 13,4 | Yes (60Gy) | GK | 8,3-35 | 17 | 70 | No | 15,1 |
| Villavicencio [55] | 2009 | retrospective | Single arm | 20 | GBM | 61.3 | 5,8 | Yes (54Gy) | CyberKnife | 12-25 | 20 | 75 | possible CT | 11,5 |
| Shrieve [56] | 1999 | retrospective | Single arm | 78 | GBM | 51 | 9,4 | Yes (60Gy) | Linac | 6-24 | 12 | 85 | No | 19,9 |
| Prisco [57] | 2002 | retrospective | 2 arms | 15 | 1 AA, 14 GBM | 51 | 15 | Yes (60Gy) | Linac | 8-12,5 | 10 | NR | No | 21,4 |
| Wang [58] | 2004 | retrospective | Single arm | 30 | GBM* | NR | 10,7 | NR | Linac | NR | 19,4 | NR | NR | 13 |
| Niranjan [59] | 2015 | retrospective | Single arm | 144 | GBM | 58 | 14 | Yes (60Gy) | GK | 9-25 | 15 | NR | NR | 8,4 |
| Larson [60] | 1996 | retrospective | 2 arms | 31 | GBM* | NR | 9,5 | Yes (NR) | GK | 5-37,5 | 16 | 50 | No | NR |
